# An Uncommon Encounter: Obstetric Management of a Bicornuate Bicollis Uterus With a Longitudinal Vaginal Septum

**DOI:** 10.7759/cureus.60645

**Published:** 2024-05-20

**Authors:** Bishnupriya Moharana, Amruta Choudhary, Anita Yadav, Neha Gangane

**Affiliations:** 1 Department of Obstetrics and Gynaecology, All India Institute of Medical Sciences, Nagpur, Nagpur, IND

**Keywords:** obstetric outcomes, ectopic pregnancy, longitudinal vaginal septum, bicornuate bicollis uterus, mullerian anomalies

## Abstract

This case report describes a rare presentation of a bicornuate bicollis uterus with a longitudinal vaginal septum in a 25-year-old woman presenting with a ruptured ectopic pregnancy. The patient's obstetric history revealed a previous cesarean section due to oligohydramnios. The diagnosis was confirmed through intraoperative assessment and MRI findings. Despite successful management of the ectopic pregnancy, the case underscores the importance of early detection and tailored management of Mullerian anomalies to optimize pregnancy outcomes. This report highlights the need for continued research to improve diagnostic accuracy and therapeutic approaches for such complex anatomical variations.

## Introduction

The merger of two paramesonephric or Mullerian ducts forms the female genital tract. The processes include septum resorption, differentiation, and morphogenesis. Thus, Mullerian anomalies represent embryological malformations of the Mullerian duct [1,2]. The prevalence of these anomalies varies, ranging from 5.5% in unselected populations to 24.5% in patients with a history of miscarriage and infertility [3]. While many patients remain asymptomatic, some present with various gynecologic and obstetric complaints such as infertility, recurrent pregnancy loss, and poor obstetric outcomes [4]. Instead of the Mllerian duct, the endoderm of the urogenital sinus forms the lower fifth of the vagina. It is not common for Mullerian abnormalities and the vaginal septum to coexist [1]. For women with uterine anomalies, imaging is crucial to the diagnosis and treatment process. Uterine anomalies are linked to unfavorable reproductive outcomes, thus, it is imperative to provide women with reproductive outcomes education and counseling, as the majority of diagnoses occur during pregnancy. The patient's presentation has a significant role in the decision of their care. This case report highlights a rare Mullerian anomaly discovered incidentally during intraoperative assessment, demonstrating different obstetric outcomes.

## Case presentation

**Figure 1:**
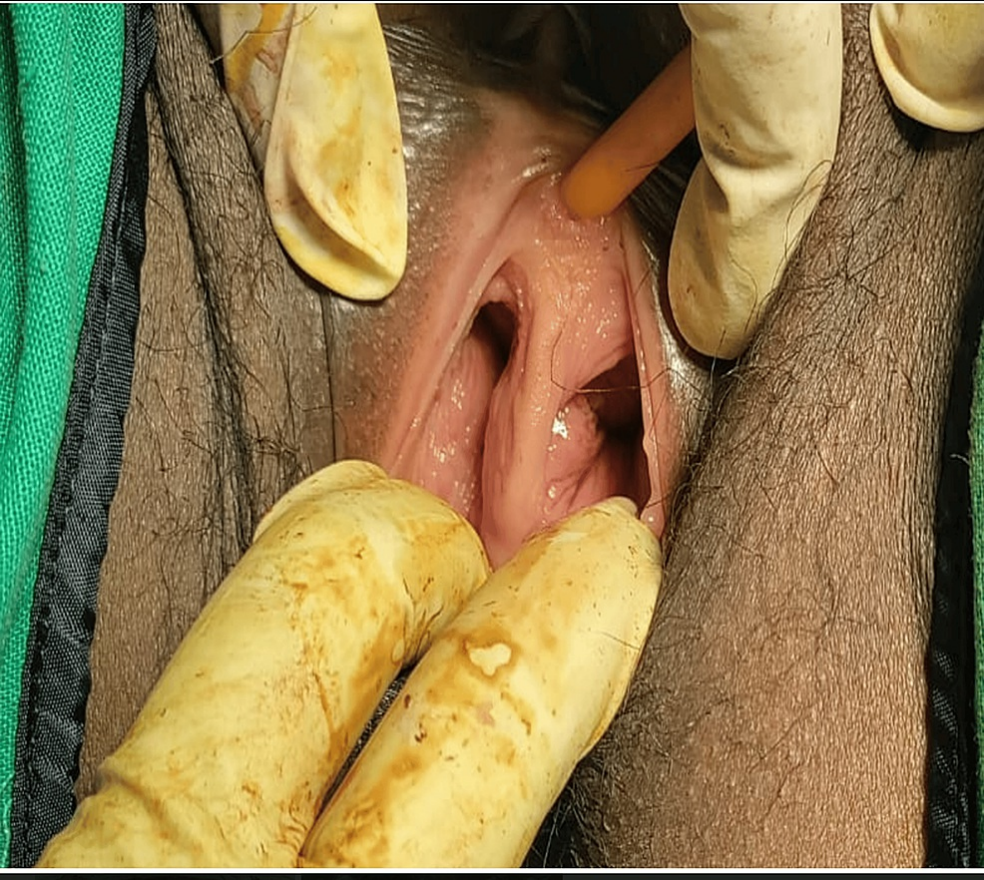
Complete longitudinal vaginal septum dividing the two vaginal cavities

**Figure 2:**
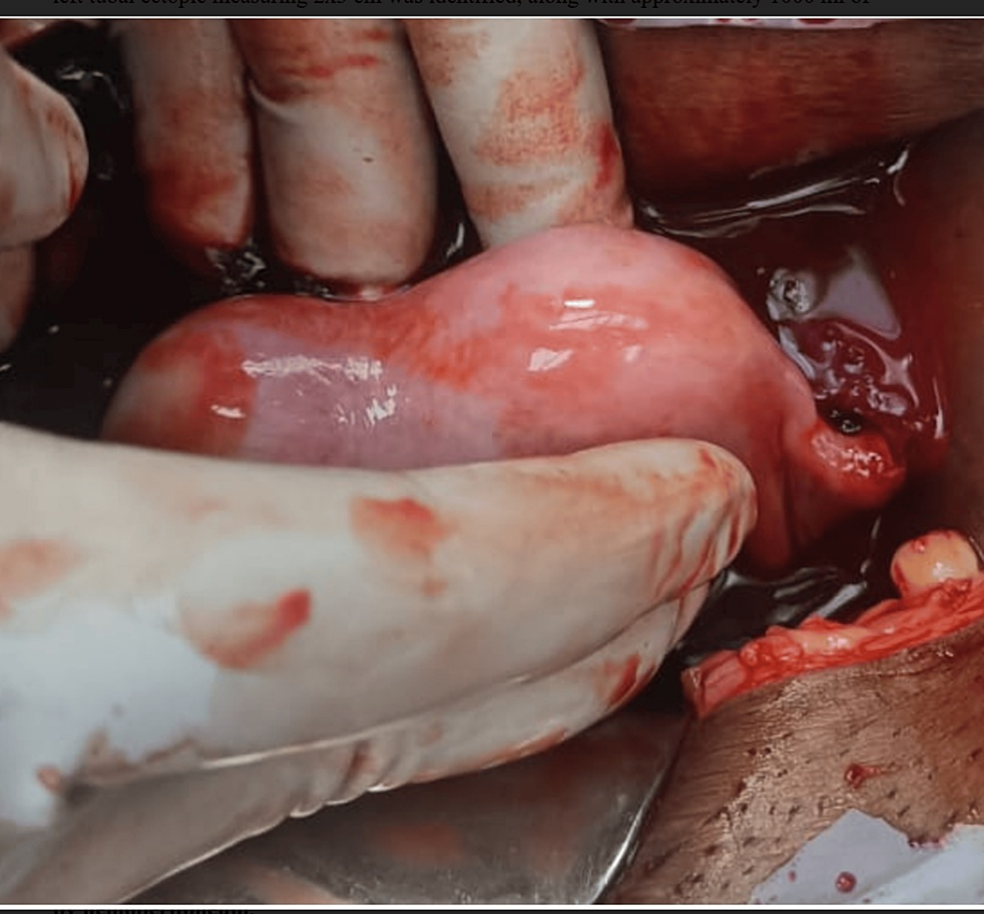
Intraoperative finding demonstrating a fundal indentation of the uterus exceeding 10 mm and ruptured ectopic pregnancy accompanied by hemoperitoneum

A 25-year-old woman, gravida 2, para 1, living child 1, presented to the emergency room with lactational amenorrhea, vaginal spotting, and abdominal pain lasting for two days. A urine pregnancy test confirmed a positive result. Her obstetric history revealed a previous spontaneous conception resulting in a cesarean section due to oligohydramnios at 36 weeks of gestation, with a healthy baby weighing 2.65 kg. Intraoperative notes from the previous cesarean section indicated the presence of a bicornuate uterus with the pregnancy located in the left cornua. Upon examination, the patient exhibited tachycardia (120 beats per minute), hypotension (100/60 mmHg), and pallor. Abdominal examination revealed diffuse tenderness while speculum examination revealed two vaginal cavities separated by intervening septa, and two cervixes, with minimal bleeding from the left cervical os (Figure 1). Per-vaginal examination revealed left fornix tenderness. Ultrasonography confirmed a ruptured left tubal ectopic pregnancy with hemoperitoneum, necessitating emergency exploratory laparotomy. Intraoperatively, a ruptured left tubal ectopic measuring 2x3 cm was identified, along with approximately 1000 ml of hemoperitoneum, leading to left salpingectomy. The uterus exhibited an arcuate shape with a fundal indentation greater than 1 cm, while the right tube and both ovaries appeared normal (Figure 2). The patient received two units of blood transfusion, and the postoperative period was uneventful.

**Figure 3:**
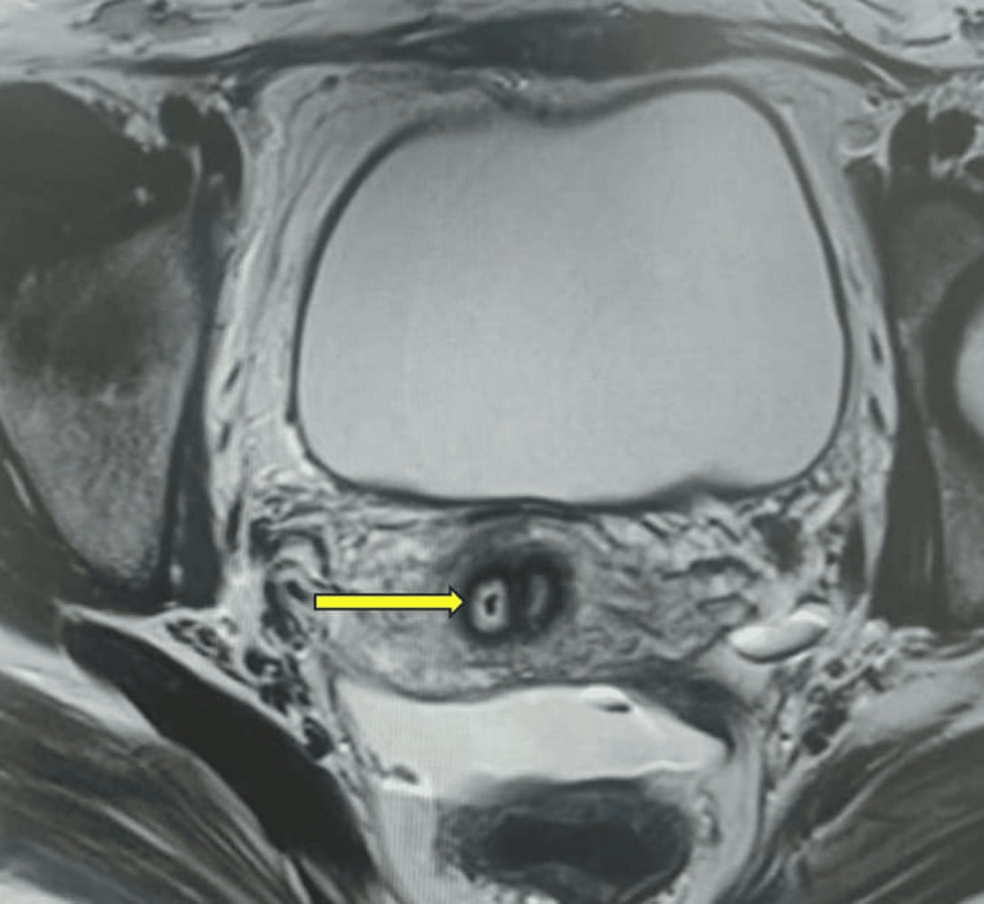
Transverse MRI section depicting a double cervix (yellow arrow points at the owl eye appearance of the double cervix on MRI)

**Figure 4:**
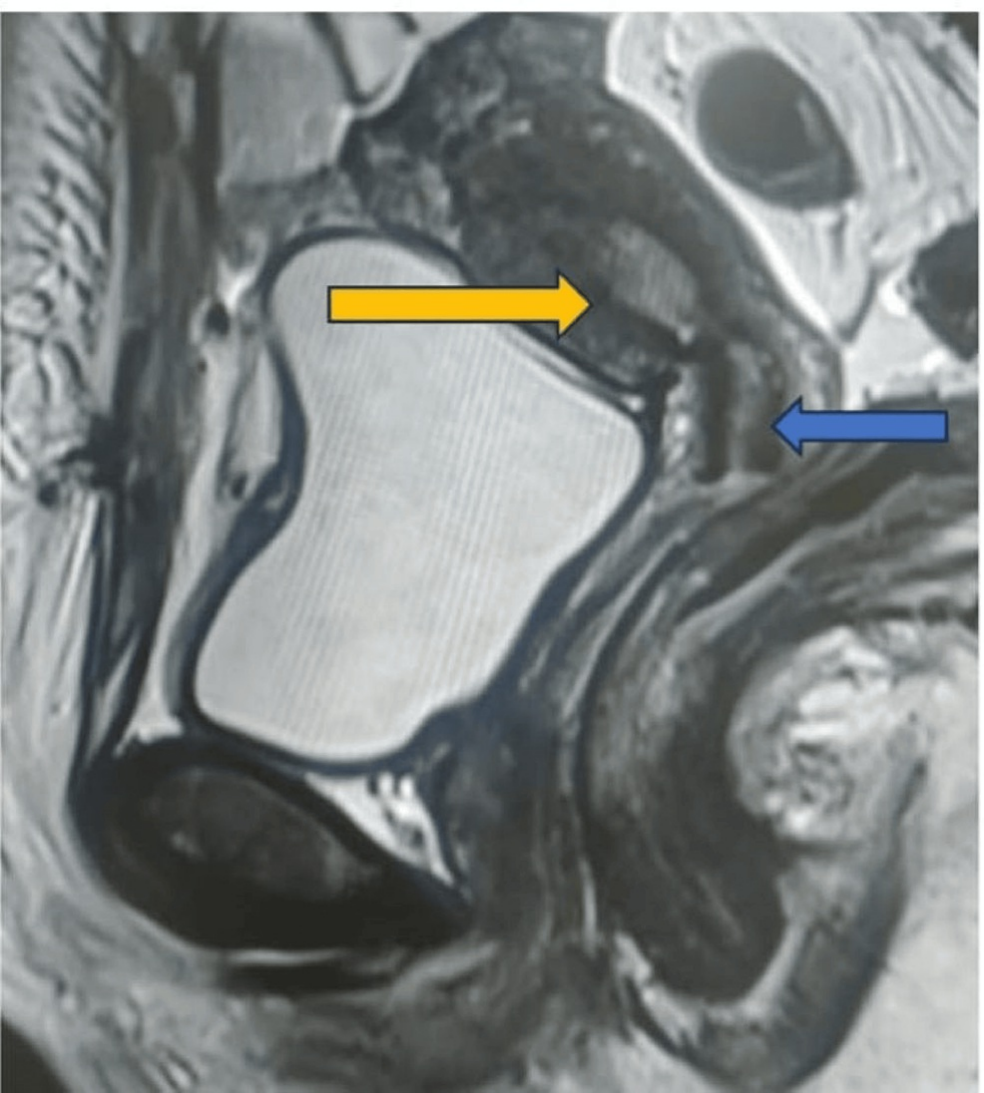
Sagittal MRI section illustrating two uterine cavities (yellow arrow: two uterine cavities, blue arrow: double cervix)

**Figure 5:**
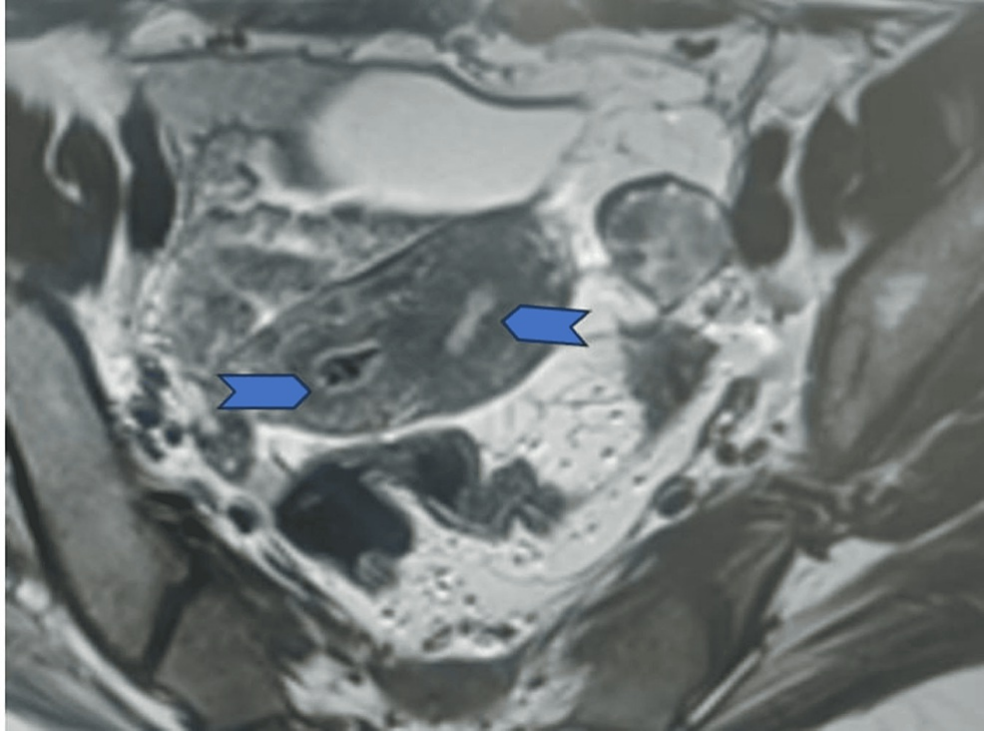
Transverse MRI section depicting double uterine cavity with intervening tissue (arrowhead: double uterine cavity)

Following surgery, an MRI revealed a mild concavity of the external uterine contour with two uterine horns, delineating two endometrial cavities extending up to the cervix, suggestive of a bicornuate bicollis uterus (Figures 3-5).

At the 15-day follow-up, the patient's vitals remained steady and she had no new symptoms. She has received counseling regarding the complications associated with uterine anomalies during pregnancy and has been advised to have a metroplasty (uterine corrective surgery) for future conception. However, she chose temporary family planning methods and does not intend to become pregnant anytime soon. An intrauterine device was placed in the right uterine horn, and she was monitored monthly for two months before being told to follow up every six months.

## Discussion

In this case, the patient exhibited a fundal indentation exceeding 10 mm, indicative of a bicornuate uterus. Additionally, per-speculum examination revealed a double cervix with a complete longitudinal vaginal septum. MRI findings supported the diagnosis of a bicornuate uterus with bicollis morphology. These observations correspond to the ESHRE (European Society of Human Reproduction and Embryology) class U3b/C2/V1 (complete bicorporeal with double normal cervix with complete non-obstructing longitudinal vaginal septum) [2] and ASRM (American Society for Reproductive Medicine) Mullerian anomalies classification 2021, categorizing the anomaly as bicornuate bicollis with complete longitudinal vaginal septum [5]. This condition can be distinguished from the septate and didelphys uterus by the presence of intervening tissue and the owl's eye appearance of two cervices on MRI.

Mullerian anomalies can arise from fusion, resorption defect, or agenesis of Mullerian ducts, resulting in various anomalies, with septate uterus being the most common (35%), followed by bicornuate uterus (26%), arcuate uterus (18%), unicornuate uterus (10%), uterus didelphys (8%), and Mullerian agenesis (3%) [6]. While many patients with Mullerian anomalies remain asymptomatic until adolescence, they may present with complications such as infertility, recurrent pregnancy loss, or pregnancy-related issues like malpresentation, preterm labor, fetal growth restriction, abnormal placentation, postpartum hemorrhage, and perinatal mortality [7,8]. The MRI is regarded as the primary screening method, followed by ultrasound, especially with a 3D probe, as a screening and diagnostic tool for the majority of Müllerian abnormalities. Other modalities are hysteroscopy and laparoscopy. The diagnostic accuracy of MRI and 3D ultrasonography for Müllerian abnormalities was similar as per the studies. When combined with more sophisticated techniques, such as 2D and 3D saline (or gel) contrast sonohysterography, 3D ultrasonography can clear up the majority of diagnostic questions regarding Müllerian defects. For the evaluation of Müllerian anomalies in all major classification systems, the coronal view is the standard plane. When compared to laparoscopy with hysteroscopy, 3D ultrasound has demonstrated great accuracy (>90%) and strong inter-rater reliability [9].

This case represents a unique presentation of a bicornuate uterus with cervical duplication and a vertical longitudinal vaginal septum, with a successful outcome in the first pregnancy. Although literature reports successful pregnancy outcomes in patients with bicornuate uterus, many are associated with complications such as previous pregnancy losses or preterm labor [10]. Notably, the patient presented with an ectopic pregnancy during lactational amenorrhea. In Mullerian anomalies, associated renal anomalies, such as ectopic kidney or renal agenesis, are common [11], but no such abnormalities were observed in our patient.

Management strategies depend on patients' symptoms like Strassman metroplasty being an option for recurrent abortions, especially when laparoscopic intervention is preferred [12]. Close follow-up is essential during pregnancy or routine evaluations to monitor for preterm labor or malpresentation. In cases detected post-successful pregnancy, intraoperative findings may necessitate close follow-up in subsequent pregnancies.

## Conclusions

The presence of Mullerian defects, and their association with pregnancy outcomes, requires further study. Early detection during routine gynecological evaluations or pregnancy is crucial for meticulous management, patient counseling, and timely intervention. Accurate diagnosis, personalized care, and ongoing monitoring are vital, highlighting the need for continued research to enhance management strategies and optimize outcomes.
